# Novel Biomarkers in Hepatocellular Carcinoma from Embryogenic Antigens to cfDNA

**DOI:** 10.3390/biomedicines13051020

**Published:** 2025-04-23

**Authors:** Robeer Ghantus, Răzvan Alexandru Ciocan, Diana Schlanger, Călin Popa, Claudia Diana Gherman, Călin Vaida, Bogdan Gherman, Doina Pîslă, Nadim Al Hajjar, Andra Ciocan

**Affiliations:** 1 Department of Internal Medicine, Bnai Zion Medical Center, Haifa 31048, Israel; roberghantous98@gmail.com; 2 Department of Surgery–Practical Abilities, “Iuliu Hațieganu” University of Medicine and Pharmacy, 400337 Cluj-Napoca, Romania; gherman.claudia@umfcluj.ro; 3 Regional Institute of Gastroenterology and Hepatology “Octavian Fodor”, 400162 Cluj-Napoca, Romania; schlanger.diana@elearn.umfcluj.ro (D.S.); cpopa@elearn.umfcluj.ro (C.P.); nadim.alhajjar@umfcluj.ro (N.A.H.); andra.ciocan@umfcluj.ro (A.C.); 4 Department of Surgery–Surgery III, “Iuliu Hațieganu” University of Medicine and Pharmacy, 400162 Cluj-Napoca, Romania; 5 “CESTER” Research Centre for the Robot Simulation and Testing, Technical University Cluj-Napoca, 400114 Cluj-Napoca, Romania; calin.vaida@mep.utcluj.ro (C.V.); bogdan.gherman@mep.utcluj.ro (B.G.); doina.pisla@mep.utcluj.ro (D.P.)

**Keywords:** hepatocellular carcinoma, embryogenic antigens, carcinoma biomarkers, alpha-fetoprotein

## Abstract

**Background:** Hepatocellular carcinoma (HCC), a primary liver cancer, continues to pose a significant challenge to the healthcare system because of its elevated incidence and fatality rates. This study aims to assess new biomarkers for early diagnosis and prognosis, comparing them to the established gold standard alpha-fetoprotein (AFP) and liver ultrasonography. **Methods:** A literature review was conducted in accordance with the preferred reporting items for systematic reviews and meta-analyses (PRISMA) guideline. A total of 670 papers were identified using internet databases. After applying the exclusion criteria, eight studies were included in this literature review. **Results:** It was identified that certain analyzed biomarkers, or the combinations thereof, exhibited superior sensitivity compared to the existing gold standard. The circulating cell-free DNA (cfDNA) and microRNAs (miRNAs), proved to have encouraging outcomes, particularly for the early identification of HCC. Additional indicators, such as circulating tumor cells (CTCs) and the alkaline phosphatase plus gamma-glutamyl transpeptidase to lymphocyte ratio (AGLR), may forecast disease progression, particularly regarding vascular invasion. **Conclusions:** These biomarkers may assist clinicians in making better therapeutical choices in order to provide personalized treatment and optimal follow-up for HCC patients.

## 1. Introduction

Hepatocellular carcinoma (HCC) is the most common primary liver tumor. The most recent statistics from the World Health Organization indicate that it was the sixth most prevalent cancer globally in 2022, with 866,136 new cases reported. HCC ranks third in cancer-related mortality, accounting for 758,725 deaths each year. Worldwide, Asia has the greatest incidence of hepatocellular carcinoma, with 607,361 new cases identified in 2022 [1].

The increasing prevalence of HCC is mostly attributable to chronic liver disease, leading to liver cirrhosis, a condition that elevates the risk of HCC more than 30 times. Hepatitis B and C contribute to 40–50% of hepatocellular carcinoma incidence [2]. In the United States, 45–55% of newly diagnosed hepatocellular carcinomas may be ascribed to hepatitis C. Additional variables, such as alcohol use, smoking, and metabolic conditions, have been identified in several studies as risk factors for hepatocellular carcinoma; however, their influence on the HCC risk is comparatively lower than that of viral hepatitis. Metabolic dysfunction-related steatosis liver disease is linked to a higher risk of liver-related complications and mortality in individuals with chronic hepatitis B. Liver steatosis can advance to metabolic dysfunction-associated steatohepatitis, such as MASH and MASLD, consequently elevating the likelihood of cirrhosis and hepatocellular carcinoma. During their lifespan, 10–29% of patients with MASH develop cirrhosis, and among these, 4–27% progress to HCC [1,2]. These factors underscore the necessity of improved early detection methods to mitigate disease progression and optimize patient outcomes.

A major challenge in the management of HCC is its late diagnosis, which significantly worsens patient outcomes. The prognosis of HCC is largely dependent on the stage at which it is diagnosed, with early detection being critical for improving survival rates. Studies have demonstrated that early-stage HCC patients who undergo curative treatments, such as surgical resection or liver transplantation, have a five-year survival rate exceeding 70%, whereas patients diagnosed in an advanced stage have a median survival of only six to twelve months [3]. Therefore, the implementation of effective screening and early diagnostic strategies is essential for reducing HCC-related mortality.

Currently, alpha-fetoprotein (AFP), a glycoprotein synthesized by the fetal liver, remains the predominant biomarker used for HCC screening and follow-up. AFP was identified in 1963 and has since become the most widely used biomarker for HCC diagnosis [4]. However, its diagnostic accuracy is suboptimal due to inadequate sensitivity and specificity [4]. Approximately 40% of HCC patients are AFP-negative, and elevated AFP levels can also occur in non-neoplastic liver diseases, such as cirrhosis, acute hepatitis, and viral hepatitis, thus limiting its reliability [5]. This necessitates the exploration of novel biomarkers that can provide superior diagnostic accuracy.

A prevalent screening technique for HCC is grey-scale ultrasonography. Nonetheless, its use is debatable, since its sensitivity is about 45% for early-stage illness, which rises to 63% when used in conjunction with AFP. A prevalent issue with ultrasound is its dependence on both the patient and the practitioner, particularly in obese individuals. Contrast-enhanced ultrasound raises its sensitivity, but its availability and the lack of personnel with expertise may limit its use [6].

Recent advances in biomarker research have introduced promising candidates for early HCC detection. The development and evolution of HCC entails intricate genetic and epigenetic modifications, during which hepatocytes may secrete tumor-associated chemicals into bodily fluids. Liquid biopsy techniques, including circulating cell-free DNA (cfDNA) and microRNAs (miRNAs), have shown potential in identifying HCC at earlier stages compared to the traditional methods. Liquid biopsy is a molecular examination of tumor by-products that may function as potential biomarkers found in blood or other body fluids [7]. Additionally, circulating tumor cells (CTCs) and the alkaline phosphatase plus gamma-glutamyl transpeptidase to lymphocyte ratio (AGLR) have demonstrated prognostic value, particularly in assessing disease progression and vascular invasion [7]. Biomarker-driven diagnostics could revolutionize HCC management by enabling non-invasive, highly sensitive screening tools that facilitate timely interventions and personalized treatment strategies.

This study aims to evaluate emerging biomarkers that may enhance HCC diagnosis, particularly in the early-stage disease. Certain biomarkers have demonstrated significant sensitivity and specificity improvements over AFP, while others show potential in predicting disease progression and patient survival. Identifying a reliable biomarker with superior diagnostic accuracy compared to current standards is critical, in order to optimize treatment strategies in patients with chronic liver disease and HCC.

## 2. Materials and Methods

This review adhered to the preferred reporting items for systematic reviews and meta-analyses (PRISMA) criteria to guarantee a meticulous and transparent methodology. Therefore, by following the PRISMA principles, a thorough and complete synthesis of the existing literature on the subject is emphasized (see Supplementary Material).

**Figure 1:**
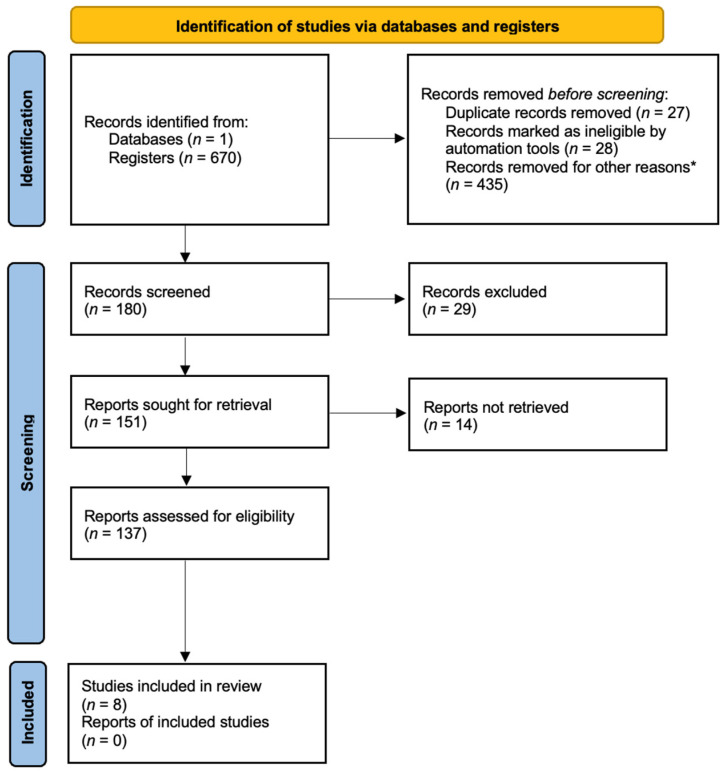
PRISMA flow diagram of the included studies and selection criteria. * The reasons for the removal of records include the following: non-English articles that did not meet the language inclusion criteria; conference abstracts, reviews, or commentaries that did not provide original research data; studies with incomplete or missing data that lacked relevant information required for systematic analysis; retracted articles that were identified during the screening process; irrelevant topics where studies were initially retrieved based on keyword searches but, upon closer inspection, did not focus on hepatocellular carcinoma biomarkers or early diagnostic methods.

The present study was designed to synthesize the new achievements in HCC diagnosis, especially early staging of the liver disease. The primary focus was on novel biomarkers with significant results compared to AFP and liver ultrasound (US), which are the current gold standards, or which could significantly aid them. To carry out this study, a systematic search of original articles, clinical trials, and experimental studies was performed using PubMed, Scopus, and Clarivate Web of Science. The search strategy was performed using the following keywords: “HCC”, “hepatocellular carcinoma”, “biomarkers”, “GPC3”, “AFP”, “cfDNA”; Mesh “Carcinoma, Hepatocellular/Diagnosis” was used as a search strategy using “AND” between keywords as a Boolean operator. All titles referred to in English and published in a determined period of time, from 2015 to 2024, were checked for eligibility by title and abstract to remove double counting. Articles referring to liver transplantation, any drug-related study, and other types of cancer were excluded. Articles on hepatocellular carcinoma diagnosis, prediction, and prognosis progress were included in the discussion. The study’s workflow is represented in Figure 1.

Two independent reviewers, R.G. and R.A.C., extracted data from the included studies. Any differences were addressed via dialogue or contact with a third reviewer (D.S.). The quality of the listed studies was evaluated using appropriate risk of bias instruments. Disagreements among the reviewers were addressed via conversation or by consulting the third reviewer.

There were 670 results found for the search strategy. Setting the limit of articles published after 2015 resulted in a total of 180 articles. Also, only full-text articles were selected, resulting in 151 articles. Finally, after applying the inclusion and exclusion criteria, 8 articles corresponded to the requirements (Figure 1).

The retrieved data were synthesized and analyzed to provide a thorough overview of the existing information regarding biomarkers in hepatocellular carcinoma. The findings are conveyed in a comprehensive fashion, without redundancy, and they are supplemented by tables and figures as necessary. This methodology was used to synthesize evidence pertinent to the research enquiries, encapsulating and elucidating the results of the included studies. The results were expressed as a percentage along with corresponding 95% confidence intervals using an exact approach for qualitative variables. The sample sizes were presented as the median and interquartile range (IQR = Q3 − Q1, where Q1 is the first quartile and Q3 is the third quartile), due to the data’s non-normal distribution (Shapiro–Wilk test). The analysis was conducted using Microsoft Excel (Version 2024).

## 3. Results

The eight studies evaluated were clinical trials; four were performed for the prognosis and prediction of HCC. The other four studies were performed to find a significant biomarker for diagnosis and early staging of HCC (Table 1).

### 3.1. Glypican-3CTC Versus AGLR and FPR

Hamaoka et al. and Liao et al. examined the use of biomarkers for predicting vascular invasion, patient outcomes, and tumor size [8,9]. Hamoaka et al. established that elevated glypican-3-positive circulating tumor cells, identified by liquid biopsy in hepatocellular carcinoma patients prior to hepatectomy, correlate with microscopic portal vein invasion (mPVI) [8]. The existence of mPVI was verified by histopathological analysis of the excised liver tissue. It also indicated that patients with CTCs of ≥5 had worse outcomes, especially reduced disease-free survival (DFS) rates and diminished overall survival (OS) rates compared to those with CTCs of <5 [8]. Liao identified an association between a high alkaline phosphatase and gamma-glutamyl transpeptidase to lymphocyte ratio (AGLR) and microvascular invasion, as determined by clinicopathological testing [9]. The research indicated that patients with an AGLR > 90 exhibited a mean OS of 59.30 months, along with 1-, 3-, and 5-year survival rates of 73.5%, 46.9%, and 36.1%, respectively (*p* < 0.001) [9]. Moreover, regarding tumor burden, both investigations indicated that the examined biomarkers correlated with increased tumor sizes. Liao et al. demonstrated that an AGLR > 90 correlated with increased tumor size (>5 cm), with a *p* value < 0.001 [9]. Hamaoka demonstrated that patients with circulating tumor cells (CTCs) of ≥5 exhibited higher tumor sizes (in mm), with a *p*-value of 0.012 [8].

Zhang et al. concentrated on predictive and prognostic indicators in hepatocellular carcinoma (HCC). The research indicated that an increased preoperative fibrinogen to prealbumin ratio (FPR) correlated with reduced recurrence-free survival (RFS) (*p* = 0.034) and OS (*p* < 0.001), with a cut-off value of 15.6 [10]. Moreover, the research revealed that an elevated FPR correlated with more advanced stages of HCC according to Barcelona Clinic Liver Cancer (BCLC) stage B/C. They discovered that patients undergoing adjuvant chemotherapy exhibited superior survival compared to those who received no adjuvant treatment, but this was only evident in the high-FPR group (*p* = 0.028). No differences were noted in the low-FPR group, although the specific chemotherapy agent evaluated was not disclosed [10]. This may assist in formulating treatment strategies for HCC patients.

### 3.2. cfDNA Versus AFP

Foda et al. and Yan et al. both illustrate the prospective use of circulating DNA in hepatocellular cancer by the analysis of plasma samples [11,12]. Foda used a machine learning model, using the DELFI technique (DNA assessment of fragments for early interception) to analyze cell-free DNA (cfDNA). The investigation revealed that HCC patients had a markedly varied fragmentation profile in comparison to the controls, non-HCC people [11]. In a high-risk group, namely individuals with cirrhosis and hepatitis, the sensitivity was 85% with a specificity of 80%. In individuals with medium risk, the sensitivity for identifying HCC was 88% with a specificity of 98% [11]. Compared to AFP, the DELFI approach demonstrated improved outcomes at every stage of HCC. Although AFP levels over 20 identified just 52% of HCC patients, DELFI was able to identify 83% of the instances that AFP overlooked. The DELFI detection rates were 79% for stage 0/A, 83% for stage B, and 95% for stage C [11]. The integration of AFP and DELFI enhanced accuracy, resulting in a combined sensitivity of 92% with a specificity of 80% [11]. Yan et al. integrated cfDNA levels with AFP and age to construct the HCC index. The results exhibited 87% sensitivity and 100% specificity at a cut-off value of 0.61 [12].

### 3.3. MFG-E8 Versus AFP and DCP

Milk fat globule-ECF factor 8 (MFG-E8) is another biomarker that surpassed AFP in performance. Shimagaki et al. examined serum MFG-E8 in hepatocellular carcinoma patients who had primary hepatectomy using the ELISA method [13]. The research indicated that MFG-E8 levels were markedly reduced in HCC patients relative to healthy volunteers (*p* < 0.0001) [13], in comparison to AFP and des-gamma-carboxyprothrombin (DCP), with cut-off values of 10 ng/mL and 40 mAU/mL, respectively. The AUC for MFG-E8 was 0.842, demonstrating a sensitivity of 69.7% and a specificity of 84.3%, which were above those of AFP and DCP [13]. Significantly, 44 HCC patients tested negative for both AFP and DCP, with 70% of these instances (31) exhibiting low levels of MFG-E8. This suggests the prospective use of MFG-E8 in instances when conventional biomarkers are inadequate [13]. The research assessed the role of MFG-E8 in the prognosis of HCC post-liver resection, revealing that patients with early recurrence, within one year after surgery, had lower serum MFG-E8 levels than those without recurrence (*p* < 0.0324). Patients experiencing early recurrence had markedly worse overall survival in comparison to both the late-recurrence cohort (1–5 years) and the no-recurrence cohort (within 5 years), with *p* < 0.0001 [13].

### 3.4. APEX1 and miRNA Versus AFP

Cao et al. examined the challenge of identifying HCC in patients with low AFP levels, particularly in relation to cirrhotic patients, and the prospective use of apyrimidinic endodeoxyribonuclease 1 (APEX1) in these instances [14]. Receiver operating characteristic (ROC) curve analysis was conducted on two datasets, GSE25097 and GSE63898, revealing AUCs of 0.7802 and 0.71, respectively (*p* < 0.0001). The AUC for AFP in these datasets was not statistically significant, as anticipated. The AFP levels in HCC cases and cirrhotic individuals were comparable [14]. Additionally, they examined the use of APEX1 in stage 1 HCC with ROC analysis, revealing an AUC of 0.80 (*p* < 0.0001), which was considerably superior to AFP for the same stage (AUC of 0.607 with *p* = 0.0252) [14]. 

Wen et al. assessed a panel of miRNAs for their potential use as biomarkers for hepatocellular carcinoma. In the screening phase of the investigation, 10 miRNAs were substantially elevated in hepatocellular carcinoma patients (*p* < 0.05) [15]. During the validation phase, four of them had significant promise as preclinical biomarkers, indicating their ability to identify cancer prior to clinical diagnosis [15]. The research integrated AFP with the four discovered miRNAs, demonstrating superior efficacy compared to the usage of AFP in isolation. In the prospective Changzhou cohort (7584 subjects aged 18–70 from Changzhou, China), this combination attained a sensitivity of 70% and a specificity of 77.5%, but in the Qidong cohort (20,000 participants aged 30–59 from Qidong, China), it acquired a sensitivity of 64% and a specificity of 83.8%. The AFP exhibited a sensitivity of 15% in the Changzhou cohort and 16% in the Qidong cohort, respectively [15].

These results underscore that novel biomarkers such as cfDNA and miRNAs may serve as promising candidates for the early diagnosis of HCC, which is particularly beneficial for high-risk groups, including individuals with chronic liver diseases like cirrhosis and chronic hepatitis.

Multiple studies [11,12,15] used non-invasive diagnostic techniques. This novel strategy may advantage patients by being less hazardous, more accessible, and cost-efficient for the detection and monitoring of hepatocellular carcinoma.

Most studies aim to find a significant biomarker with relevant specificity and sensitivity; these values are calculated both alone and in contrast to AFP (Table 2).

Two studies were interested in calculating the ratio between the usual biochemical blood analyses for a better preoperative and postoperative prognosis and for a better management of systemic therapy necessity. Zhang et al. [10] showed that a high FPR was significantly associated with decreased recurrence-free survival (*p* < 0.05) and overall survival (*p* < 0.001) within HCC patients. Also, they observed that the survival of surgical HCC patients receiving chemotherapy was significantly longer than that of the patients without the treatment in a high-FPR subgroup (*p* = 0.028); this was not observed in the low-FPR subgroup, although they did not mention the chemotherapy agent that was evaluated between the previously used tyrosine-kinase inhibitors and the newly introduced immunotherapy combination with atezolizumab-bevacizumab. Liao et al. [9] aimed to investigate the relationship between the AGLR and the progression as well as the prognosis of HCC. The elevated preoperative AGLR level (>90) indicated poor prognosis for patients with HCC.

Two other studies focused on cell-free DNA levels and fragmentomes for hepatocellular carcinoma diagnosis. Foda et al. (2023) [11], using a machine learning model that incorporated multifeatured fragmentome data from 724 individuals, detected hepatocellular carcinoma with high sensitivity and specificity in average and high-risk populations, including in early-stage disease. Yan et al. (2018) [12] showed that their results suggest that the combination of cfDNA with age and AFP could improve the diagnostic performance for HCC. The HCC index, a combination model including age, cfDNA, and AFP, was used.

## 4. Discussion

Hepatocellular carcinoma is the primary cause of mortality in individuals with liver cirrhosis. A significant problem in managing HCC is its late identification, resulting in unfavorable patient outcomes. Alpha-fetoprotein, identified more than 60 years ago, is the most used biomarker for the screening, diagnosis, prognostication, and assessment of treatment for hepatocellular carcinoma. Nevertheless, owing to its comparatively inadequate sensitivity, contemporary research is exploring other novel biomarkers for the therapy of HCC [16].

A novel suggested instrument for the identification of HCC is the use of liquid biopsy to detect circulating cell-free DNA, which is beginning to be integrated into clinical practice. Circulating cfDNA comprises the portion of cfDNA originating from cancer cells, mostly consisting of 160–189 base pair fragments primarily produced by apoptotic cells. As a non-invasive instrument, it can monitor the progression of certain genetic alterations associated with diseases, including HCC, therefore presenting a potentially important resource for HCC diagnosis [17].

Jiang et al. used massively parallel sequencing to analyze the size distribution of plasma DNA in 90 individuals with hepatocellular cancer. The research indicated a disparity in plasma DNA size between HCC patients and healthy controls [18]. The plasma DNA in all the groups had a peak at 166 base pairs, indicating an apoptotic origin, but the HCC patients presented an extra population of both short and long DNA fragments. The researchers then devised a method termed CAZA (chromosome arm-level z-score analysis) to differentiate tumor-derived DNA from non-tumor-derived DNA, revealing that the tumor-derived DNA was shorter than the non-tumoral DNA [18].

The included research demonstrated the efficacy of circulating cfDNA, since Foda et al. discovered that individuals with HCC had a very varied fragmentation profile. Yan et al. developed the HCC index by integrating cfDNA with AFP and age, yielding encouraging outcomes [11].

Another use of liquid biopsy in hepatocellular carcinoma is the isolation of circulating tumor cells. Kelley et al. sought to assess the viability of identifying and isolating circulating tumor cells in patients with hepatocellular carcinoma. Twenty patients with metastatic HCC and ten with non-malignant liver disease NMLD were included in the research and CTCs were detected using the epithelial cell adhesion molecule (EpCAM). The research established that circulating EpCAM-positive cells are seldom seen in non-malignant liver disorders and that their presence generally signifies a malignant etiology [19]. Moreover, the research indicated that the identification of ≥1 CTC/7.5 mL correlated with the occurrence of vascular invasion (*p* = 0.009) [19]. Hamaoka et al. demonstrated that circulating tumor cells (CTCs) of ≥5 correlated with worse patient outcomes; however, the marker used for CTC identification was glypican-3 rather than EpCAM [8].

MicroRNAs are small non-coding RNAs that act as gene regulators that lead to miRNA degradation, many of which have been found to be involved in the cell cycle of HCC by mediating cell proliferation and apoptosis [20]. Zhu et al. found that miR-10b was highly expressed in HCC tissue and that the overexpression enhanced the HCC cell migration and invasion [21]. Wang et al. revealed that miR-25 can stimulate the growth of HCC cells and activates the epithelial–mesenchymal transition (EMT) [22]. Among the reviewed studies, Wen et al. found four miRNAs, miR-20a-5p, miR-320a, miR-324-3p, and miR-375, that could be used as preclinical biomarkers [15].

Xie et al. discovered that miR-320a may act as a tumor suppressor by targeting the c-Myc oncogene, thereby suppressing cancer cell proliferation [23]. Xue et al. tackled the issue of managing multidrug-resistant hepatocellular carcinoma, which is particularly attributable to the overexpression of P-glycoprotein (P-gp). The research demonstrated that miR-375 inhibits P-glycoprotein, and when administered with doxorubicin hydrochloride (DOX), it mitigates drug efflux [24].

Microvascular invasion (MVI) is associated with early tumor recurrence and worse overall patient outcomes. MVI was shown to be a more significant predictor of recurrence than the Milan criteria for HCC [25]. Given its significant influence on survival, several studies are examining certain characteristics that may forecast MVI in HCC. Hirokawa et al. demonstrated that positive L3-AFPm PIVKA-II > 150 mAU/mL and preoperative tumor size ≥ 3 cm correlate with positive MVI [26]. Liao et al. [9] demonstrated an association between an elevated AGLR and MVA; moreover, they identified that an AGLR > 90 correlates with greater tumor size (>5), which, according to Hirokawa, predicts MVI [26].

Other investigations focused on forecasting the prognosis of patients with HCC. Mai et al. examined various inflammatory-based models for this purpose. The research indicated that an elevated fibrinogen to albumin ratio correlates with decreased overall survival and disease-free survival [27]. Zhang et al. similarly discovered that an increased preoperative fibrinogen to prealbumin ratio correlated with worse recurrence-free survival and overall survival [10]. These inflammatory-based models may assist in clinical decision making and therapy alternatives.

Research is underway to identify enzymes implicated in DNA base excision repair, which is associated with several malignancies. Sun et al. investigated the function of APEX1, a DNA repair enzyme which is implicated in HCC development [28]. The research revealed that APEX1 is overexpressed in hepatocellular carcinoma tissues, and its elevated expression is associated with reduced 5-year OS. This work showed that suppressing APEX1 results in decreased cell proliferation, reduced invasion, and heightened apoptosis [28]. Cao et al. found in their review that APEX1 expression escalated with advancing tumor grades and stages and emphasized the significance of APEX1, particularly in patients with low AFP levels [14].

Ko et al. examined the function of milk fat globule factor 8 in the advancement and proliferation of hepatocellular carcinoma tissues. The research indicated that HCC samples had elevated MFG-E8 expression relative to normal tissues [29]. In vitro studies showed that the overexpression of MFG-E8 enhanced the proliferation and migration of HCC cells [29]. In contrast, Shimigaki et al. observed that blood MFG-E8 levels were decreased in HCC patients, indicating its potential use as a diagnostic biomarker [13].

The development of biomarkers is closely linked to advancements in detection technologies, which have significantly improved the sensitivity and specificity of cancer diagnostics. One notable innovation is microfluidics technology, which has played a pivotal role in the refinement of circulating tumor cell technology. Microfluidic platforms enable the precise isolation, enrichment, and analysis of CTCs from blood samples, facilitating a minimally invasive approach to early cancer detection and monitoring. In the context of hepatocellular carcinoma, these advancements enhance the ability to detect tumor progression and metastasis at earlier stages, potentially improving patient outcomes [30,31]. As CTC technology continues to evolve, it is expected to contribute to the development of personalized treatment strategies by allowing real-time monitoring of tumor dynamics and therapeutic response.

## 5. Conclusions

The present study emphasizes the significant burden of hepatocellular carcinoma upon the medical system and the urgent need for novel instruments of early detection and prognosis of disease progression. Recently discovered biomarkers were analyzed in terms of sensitivity and specificity in an attempt to surpass the current screening gold standard, which continues to rely on AFP and US. CfDNA fragmentomes express a specificity of 98% for an AFP cut-off >20 ng/mL compared to cfDNA alone at 93.6% for >80.5 ng/mL, and miRNA showed a sensitivity of 86.6% compared to serum milk fat globule-EGF factor 8 with a DCP and APEX of 81.1%.

## Figures and Tables

**Table 1:** Purpose and biomarker names of the investigated studies.

Author	Biomarker	Cases (Numbers)	Purpose
Hamaoka [8]	CTC-GPC3	85	Prognostic and Metastases
Liao [9]	AGLR	495	Progression and Prognosis
Zhang [10]	FPR	230	Prediction and Prognosis
Foda [11]	cfDNA fragmentome	724	Early staging and Diagnosis
Yan [12]	cfDNA	86	Early staging and Diagnosis
Shimagaki [13]	MFG-E8	282	Diagnosis and Prognosis
Cao [14]	APEX1	45	Diagnosis and Prognosis
Wen [15]	miRNAs	149	Early staging and Diagnosis

CTC (circulating tumor cells); GPC3 (glypican-3); AGLR (alkaline phosphatase + gamma-glutamyl transpeptidase)/lymphocyte ratio; cfDNA (cell-free DNA); FPR (fibrinogen to prealbumin ratio); MFG-E8 (milk fat globule-EGF factor 8); miRNAs (microRNAs); APEX 1 (apyrimidinic endodeoxyribonuclease 1).

**Table 2:** Biomarkers with higher sensitivity and specificity than AFP in their studies.

Studies	Sensitivity	Specificity	AFP Cut-off Used (ng/mL)
Cell-Free DNA Fragmentomes (average risk)	88%	98%	>20
Cell-Free DNA Fragmentomes (high risk)	85%	80%	>20
Circulating cell-free DNA + age + AFP (HCC index)	87%	100%	>80.5
Circulating cell-free DNA alone	62.5%	93.6%	>80.5
Serum Milk Fat Globule-EGF factor 8 + DCP	81.1%	89.8%	>10
miRNA panel	86.6%	64.6%	>400
APEX1	n.a.	n.a.	n.a.

DNA (deoxyribonucleic acid); EGF factor 8 (epidermal growth factor 8); DCP (des-gamma-carboxyprothrombin); miRNA (microribonucleic acid); APEX 1 (apyrimidinic endodeoxyribonuclease 1); n.a. (not available).

## Data Availability

Data fully available on demand from the corresponding author.

## Supplementary Materials

The following supporting information can be downloaded at: https://www.mdpi.com/article/10.3390/biomedicines13051020/s1, PRISMA 2020 Checklist.

## References

[1] (2024). Cancer Today. https://gco.iarc.fr/today/en/fact-sheets-cancers.

[2] Ayada I., Li J., Brouwer W.P., de Knegt R.J., Pan Q. (2024). Impact of chronic hepatitis B and concurrent steatosis on the risk of hepatocellular carcinoma. Hepatol. Int..

[3] Piñero F., Dirchwolf M., Pessôa M.G. (2020). Biomarkers in Hepatocellular Carcinoma: Diagnosis, Prognosis and Treatment Response Assessment. Cells.

[4] Zhou J.-M., Wang T., Zhang K.-H. (2021). AFP-L3 for the Diagnosis of Early Hepatocellular Carcinoma. Medicine.

[5] Hanif H., Ali M.J., Susheela A.T., Khan I.W., Luna-Cuadros M.A., Khan M.M., Lau D.T.-Y. (2022). Update on the Applications and Limitations of Alpha-Fetoprotein for Hepatocellular Carcinoma. World J. Gastroenterol..

[6] Singal A.G., Tayob N., Singal A.G. (2022). Blood-Based Biomarkers for Hepatocellular Carcinoma Screening: Approaching the End of the Ultrasound Era?. J. Hepatol..

[7] Pelizzaro F., Cardin R., Penzo B., Pinto E., Vitale A., Cillo U., Russo F.P., Farinati F. (2021). Liquid Biopsy in Hepatocellular Carcinoma: Where Are We Now?. Cancers.

[8] Hamaoka M., Kobayashi T., Tanaka Y., Mashima H., Ohdan H. (2019). Clinical significance of glypican-3-positive circulating tumor cells of hepatocellular carcinoma patients: A prospective study. PLoS ONE.

[9] Liao Y., Wei R., Yao R., Qin L., Li J., Yu J., Liao W. (2021). AGLR is a novel index for the prognosis of hepatocellular carcinoma patients: A retrospective study. BMC Surg..

[10] Zhang L., Chen Q.-G., Li S.-Q., Zhang J., Min Q.-H., Gao Q.-F., Sun F., Jiang Y.-H., Wang X.-Z., Ying H.-Q. (2018). Preoperative fibrinogen to prealbumin ratio as a novel predictor for clinical outcome of hepatocellular carcinoma. Future Oncol..

[11] Foda Z.H., Annapragada A.V., Boyapati K., Bruhm D.C., Vulpescu N.A., Medina J.E., Mathios D., Cristiano S., Niknafs N., Luu H.T. (2023). Detecting liver cancer using Cell-Free DNA fragmentomes. Cancer Discov..

[12] Yan L., Chen Y., Zhou J., Zhao H., Zhang H., Wang G. (2018). Diagnostic value of circulating cell-free DNA levels for hepatocellular carcinoma. Int. J. Infect. Dis..

[13] Shimagaki T., Yoshio S., Kawai H., Sakamoto Y., Doi H., Matsuda M., Mori T., Osawa Y., Fukai M., Yoshida T. (2019). Serum milk fat globule-EGF factor 8 (MFG-E8) as a diagnostic and prognostic biomarker in patients with hepatocellular carcinoma. Sci. Rep..

[14] Cao L., Cheng H., Jiang Q., Li H., Wu Z. (2020). APEX1 is a novel diagnostic and prognostic biomarker for hepatocellular carcinoma. Aging.

[15] Wen Y., Han J., Chen J., Dong J., Xia Y., Liu J., Jiang Y., Dai J., Lu J., Jin G. (2015). Plasma miRNAs as early biomarkers for detecting hepatocellular carcinoma. Int. J. Cancer.

[16] Hu X., Chen R., Wei Q., Xu X. (2021). The landscape of alpha fetoprotein in hepatocellular carcinoma: Where are we?. Int. J. Biol. Sci..

[17] Ng C.K.Y., Di Costanzo G.G., Terracciano L.M., Piscuoglio S. (2018). Circulating Cell-Free DNA in Hepatocellular Carcinoma: Current insights and outlook. Front. Med..

[18] Jiang P., Chan C.W.M., Chan K.C.A., Cheng S.H., Wong J., Wong V.W.-S., Wong G.L.H., Chan S.L., Mok T.S.K., Chan H.L.Y. (2015). Lengthening and shortening of plasma DNA in hepatocellular carcinoma patients. Proc. Natl. Acad. Sci. USA.

[19] Kelley R.K., Magbanua M.J.M., Butler T.M., Collisson E.A., Hwang J., Sidiropoulos N., Evason K., McWhirter R.M., Hameed B., Wayne E.M. (2015). Circulating tumor cells in hepatocellular carcinoma: A pilot study of detection, enumeration, and next-generation sequencing in cases and controls. BMC Cancer.

[20] Xu X., Tao Y., Shan L., Chen R., Jiang H., Qian Z., Cai F., Ma L., Yu Y. (2018). The role of MicroRNAs in hepatocellular carcinoma. J. Cancer.

[21] Zhu Q., Gong L., Wang J., Tu Q., Yao L., Zhang J.-R., Han X.-J., Zhu S.-J., Wang S.-M., Li Y.-H. (2016). miR-10b exerts oncogenic activity in human hepatocellular carcinoma cells by targeting expression of CUB and sushi multiple domains 1 (CSMD1). BMC Cancer.

[22] Wang C., Wang X., Su Z., Fei H., Liu X., Pan Q. (2015). miR-25 promotes hepatocellular carcinoma cell growth, migration and invasion by inhibiting RhoGDI1. Oncotarget.

[23] Xie F., Yuan Y., Xie L., Ran P., Xiang X., Huang Q., Qi G., Guo X., Xiao C., Zheng S. (2017). miRNA-320a inhibits tumor proliferation and invasion by targeting c-Myc in human hepatocellular carcinoma. OncoTargets Ther..

[24] Xue H., Yu Z., Liu Y., Yuan W., Yang T., You J., He X., Lee R.J., Li L., Xu C. (2017). Delivery of miR-375 and doxorubicin hydrochloride by lipid-coated hollow mesoporous silica nanoparticles to overcome multiple drug resistance in hepatocellular carcinoma. Int. J. Nanomed..

[25] Lim K.-C., Chow P.K.-H., Allen J.C., Chia G.-S., Lim M., Cheow P.-C., Chung A.Y.F., Ooi L.L.P., Tan S.-B. (2011). Microvascular invasion is a better predictor of tumor recurrence and overall survival following surgical resection for hepatocellular carcinoma compared to the Milan criteria. Ann. Surg..

[26] Hirokawa F., Hayashi M., Miyamoto Y., Asakuma M., Shimizu T., Komeda K., Inoue Y., Uchiyama K. (2013). Outcomes and predictors of microvascular invasion of solitary hepatocellular carcinoma. Hepatol. Res..

[27] Mai R.-Y., Bai T., Luo X.-L., Wu G.-B. (2022). Preoperative fibrinogen-to-albumin ratio predicts the prognosis of patients with hepatocellular carcinoma subjected to hepatectomy. BMC Gastroenterol..

[28] Sun Z., Chen G., Wang L., Sang Q., Xu G., Zhang N. (2022). APEX1 promotes the oncogenicity of hepatocellular carcinoma via regulation of MAP2K6. Aging.

[29] Ko D.S., Kim S.H., Park J.Y., Lee G., Kim H.J., Kim G., Chi K.Y., Kim I., Lee J., Won K.-Y. (2020). Milk fat Globule-EGF factor 8 contributes to progression of hepatocellular carcinoma. Cancers.

[30] Descamps L., Le Roy D., Deman A.-L. (2022). Microfluidic-Based Technologies for CTC Isolation: A Review of 10 Years of Intense Efforts towards Liquid Biopsy. Int. J. Mol. Sci..

[31] Pisla D., Calin V., Birlescu I., Hajjar N.A., Gherman B., Radu C., Plitea N. (2020). Risk Management for the Reliability of Robotic Assisted Treatment of Non-resectable Liver Tumors. Appl. Sci..

